# A method with ultra-high angular resolution for X-ray diffraction experiments

**DOI:** 10.1107/S160057752300961X

**Published:** 2024-01-01

**Authors:** X. M. Zhang, X. Zheng, X. L. Li, F. Q. Meng, S. S. Yin

**Affiliations:** a Institute of Advanced Science Facilities, Shenzhen 518107, People’s Republic of China; b Shanghai Advanced Research Institute, Chinese Academy of Sciences, Shanghai 201204, People’s Republic of China; c Sun Yat-sen University, Guangzhou 510275, People’s Republic of China; d ShanghaiTech University, Shanghai 201210, People’s Republic of China; Paul Scherrer Institut, Switzerland

**Keywords:** deconvolution, super resolution, X-ray diffraction, crystal truncation rod

## Abstract

In X-ray diffraction measurements, the angular resolution has a detection limit due to the receiving size of the detector. In many cases this detection limit is too large and must be breached to obtain the desired information. A novel method is proposed here by making the detector simultaneously measuring and moving. Using the deconvolution algorithm to remove the convolution effect, the pixel size limitation is finally broken. The algorithm used is not a common one, and suppresses signals at high frequencies, ensuring the reliability of the peak shape after restoration. The feasibility of this method is verified by successfully measuring the crystal truncation rod signal of SrTiO_3_ single crystal, and the resolution is nearly ten times higher than that of a single pixel. Moreover, this method greatly reduces the noise and improves the signal-to-noise ratio.

## Abstract 2

A method to acquire X-ray scattered signals at high frequency while the detector is moving is proposed. The algorithm provided can break through the pixel size limitation, and the resolution is improved by almost ten times compared with that of a single pixel.

## 1. Introduction

In X-ray diffraction measurements, there are several parameters that will affect the angular resolution of the tested data, for example the size of the sample and the receiving size of the detector. The receiving size has an intuitive impact on the angular resolution and leads to a detection limit. For point detectors the size of the receiving slit determines this limit, while for line detectors it is the width of the channel, and for area detectors the influencing factor is the pixel size. Larger pixel sizes cause lower resolution, which means that the detector’s ability to capture the spatial distribution of X-ray information is poorer. This will lead to blur and distortion of X-ray images, and affect the quality and reliability of the data. Common solutions are reducing the size of the receiving slit, using crystal analyzers (for point detectors) and increasing the distance between the detector and the sample (Habib *et al.*, 2018 ▸; Egan *et al.*, 2010 ▸; Thanakitivirul *et al.*, 2019 ▸; Disselhorst-Klug *et al.*, 1997 ▸; Sprigg *et al.*, 2016 ▸; Gozzo *et al.*, 2004 ▸). However, reducing the size of the receiving slit will reduce the intensity of the signal, extend the measurement time and reduce the quality of the signal. On the one hand, adjusting the distance between the detector and sample is determined by the spatial layout of the facility and cannot be adjusted arbitrarily. On the other hand, due to the limited adjustment distance, the enhancement in resolution usually cannot meet the requirements. An alternative method is moving the detector and reducing the motion step of the detector so that the angle of a single step is less than the angle occupied by the pixel/channel. In this case, the theoretical minimum resolution will depend on the mechanical minimum motion step size. Moreover, the obtained result contains the physical signal and geometric broadening of the pixel/channel, and deconvolution is necessary to obtain the accurate physical signal. However, for hybrid pixel detectors, the pixel/channel geometric broadening is a box filter, and its Fourier transform corresponds to a sinc function whose modulus will be close to zero in many places. If the inverse filter is calculated directly according to the convolution theorem, the result will be corrupted by noise. In a word, this deconvolution process is complex and needs to be carried out in a suitable way.

## 2. Related works

In the presence of convolution, the X-ray detection signal has the following relationship with the physical signal,

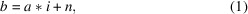

where *i* is the physical signal of the structure of the tested material, *a* is the convolution kernel, *n* is the noise, *b* is the signal obtained by the detector, and the symbol * represents the convolution operation. If there is no noise term, then, according to the convolution theorem, 

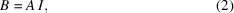

where *B*, *A* and *I* are the Fourier transforms of *b*, *a* and *i*, respectively. In theory, *I* = *B*/*A*, and *I* can be obtained directly with the inverse Fourier transform of *I*, and then the physical signal representing the material structure can be obtained; this method is called direct inverse filtering. However, due to the existence of noise, according to the linear property of the Fourier transform, the Fourier transform term of the noise *N* must also be included in *B*; the Fourier transform term of the noise has high intensity at both low and high frequencies, whereas the signal commonly only has high intensity at low frequencies. Direct inverse filtering will cause the final result to be dominated by noise and cannot reconstruct the original signal *i* reliably. In order to solve this problem, a variety of algorithms have been developed, including the Wiener filtering algorithm (Reddy & Jayaraman, 2019 ▸; Olivo *et al.*, 2000 ▸), least-squares filtering algorithm (Shruthi & Satheeshkumar, 2017 ▸), geometric mean-square filtering algorithm (Suman *et al.*, 2014 ▸), total variational algorithm (Perrone & Favaro, 2014 ▸) and continuous Fourier transform algorithm (Wang *et al.*, 2023 ▸). The basic principle of these algorithms is to suppress degraded signals at high frequencies, but there are also some problems with these algorithms. Because the high-frequency signal is directly suppressed, the final signal is mainly dominated by the low-frequency signal, so there are oscillations in some areas where the signal intensity changes rapidly, called ringing artifacts in 2D results and Gibbs oscillations in 1D results. In traditional image processing, such artifacts are sometimes acceptable, having limited impact on the overall result. In spectroscopy, however, which concerns the intensity of rays, such oscillations are of course unacceptable. When Gibbs oscillations occur on both sides of the X-ray diffraction peak, the intensity of the diffraction peak will decrease, resulting in a loss of relevant important information, and the Gibbs oscillations on both sides can easily be confused with nearby weak peaks. In order to better restore the original signal, many iterative algorithms have been developed.

The golden deconvolution algorithm (Zhou *et al.*, 2017 ▸; Morháč *et al.*, 1997 ▸, 2002 ▸) is a nonlinear iterative algorithm based on matrix formulas, originally developed for nuclear physics counting experiments. It performs denoising processing on restored signals to improve the quality of the final result, and has made some advancements compared with previous methods: firstly, it ensures the non-negativity of the computed results; additionally, it greatly reduces oscillations, particularly around sharp peaks.

The Richardson–Lucy algorithm (Zhang *et al.*, 2019 ▸; Li *et al.*, 2008 ▸; Zha *et al.*, 2014 ▸) is an iterative technique for image restoration under a Poisson noise background. It is designed to maximize the likelihood of recovered images by using an expectation maximization algorithm. The algorithm needs to estimate the process of image degradation well in order to achieve accurate restoration. Therefore, many acceleration algorithms have appeared, among them the method based on the vector extrapolation principle proposed by Biggs & Andrews (1997 ▸), showing important improvements in speed and stability.

The basic idea of the MAP algorithm (Shen *et al.*, 2004 ▸; Ng & Yip, 2001 ▸) is to use the Bayesian formula to calculate the posterior probability distribution of the original image of a given fuzzy image. It then uses this distribution to calculate the most likely original image.

Compared with the above algorithms, the results of these kinds of iterative algorithm are often better, but the simulation data results show that there are still oscillations near the diffraction peak. In addition, because of the use of iteration, the convergence rate will be slower. Other traditional algorithms and artificial intelligence algorithms (Xu *et al.*, 2021 ▸; Yang & Ji, 2019 ▸; Ren *et al.*, 2020 ▸; Li *et al.*, 2022 ▸) are also developing vigorously, but their availability and effectiveness remain to be tested.

Faced with the above problems, researchers have put forward some solutions. Raskar *et al.* (2006 ▸) proposed that the shutter can be encoded so that the convolution window is no longer a box filter but a series of box filters of varying widths, so that the Fourier transformation of such filter is no longer a sinc function. They designed a code where the modulus of the corresponding filter’s Fourier transformation does not show deep dips or zeros, as often exists for box filters, and the whole Fourier transformation modulus curve is above that of the box filters. Finally, a good result is obtained. However, this technique is not very suitable for transplanting to XRD experiments.

Agrawal *et al.* (2009 ▸) proposed an algorithm to reconstruct the original signal with several box filters of different widths. Although the Fourier transform of each box filter is a sinc function, with spikes whose amplitudes are close to zero, the effect of the combined three box filters is much more stable, and does not have any zeros. This algorithm requires that there is no integer multiple relationship between the width of these box filters. This algorithm has achieved very good results and also eliminates oscillations. Although it cannot be directly transplanted to XRD experiments, it provides a way of combining different filters to remove the zero points that exist after Fourier transformation.

## 3. Proposed algorithm

### 3.1. A detector moving at constant speed while collecting at high frequency is equivalent to a trapezoidal filter

**Figure 1:**
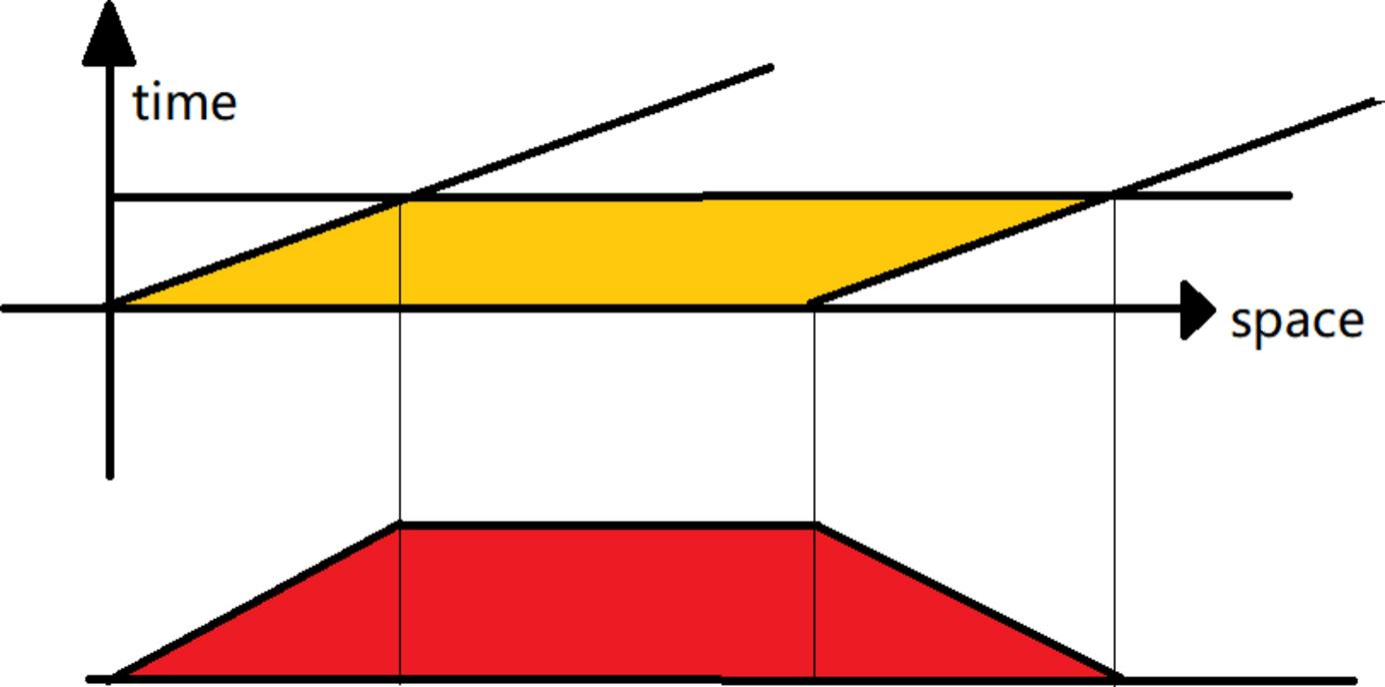
Schematic diagram of equivalent filtering of signals collected while the detector is moving.

Firstly, the spatial orientation of the sample is adjusted, and then the Eiger 500K detector is adjusted to a certain position of the reciprocal rod. The maximum sampling frequency of the Eiger 500K detector at BL02U2 at Shanghai Radiation Synchrotron Facility (SSRF) can reach up to 3000 frames s^−1^, so the scattered signal will sweep through a set of pixels on the detector when rotating the arm of the diffractometer while collecting the signal at high frequency. For each frame an angle–intensity curve can be calculated, so many angle–intensity curves can be obtained from these frames. The final angle–intensity curve can be obtained by adding the intensities at the same scattering angle of these curves together, which can play the role of noise suppression in the first step. After the noise-suppressed signal is obtained, subsequent reconstruction processing can begin. As shown in Fig. 1 ▸, the width of the yellow area represents the width of a pixel, and the height represents the time of pixel movement. When combined with the original signal, it can be seen that the result obtained is equivalent to the convolution of the original scattered signal with a trapezoidal filter (red region in Fig. 1 ▸). The upper and lower width of this trapezoid is determined by the pixel size and the spatial range of the pixel movement during the acquisition time. By changing the rotational velocity of the arm of the diffractometer, different trapezoidal filters can be obtained.

### 3.2. Three-trapezoidal filter deconvolution

**Figure 2:**
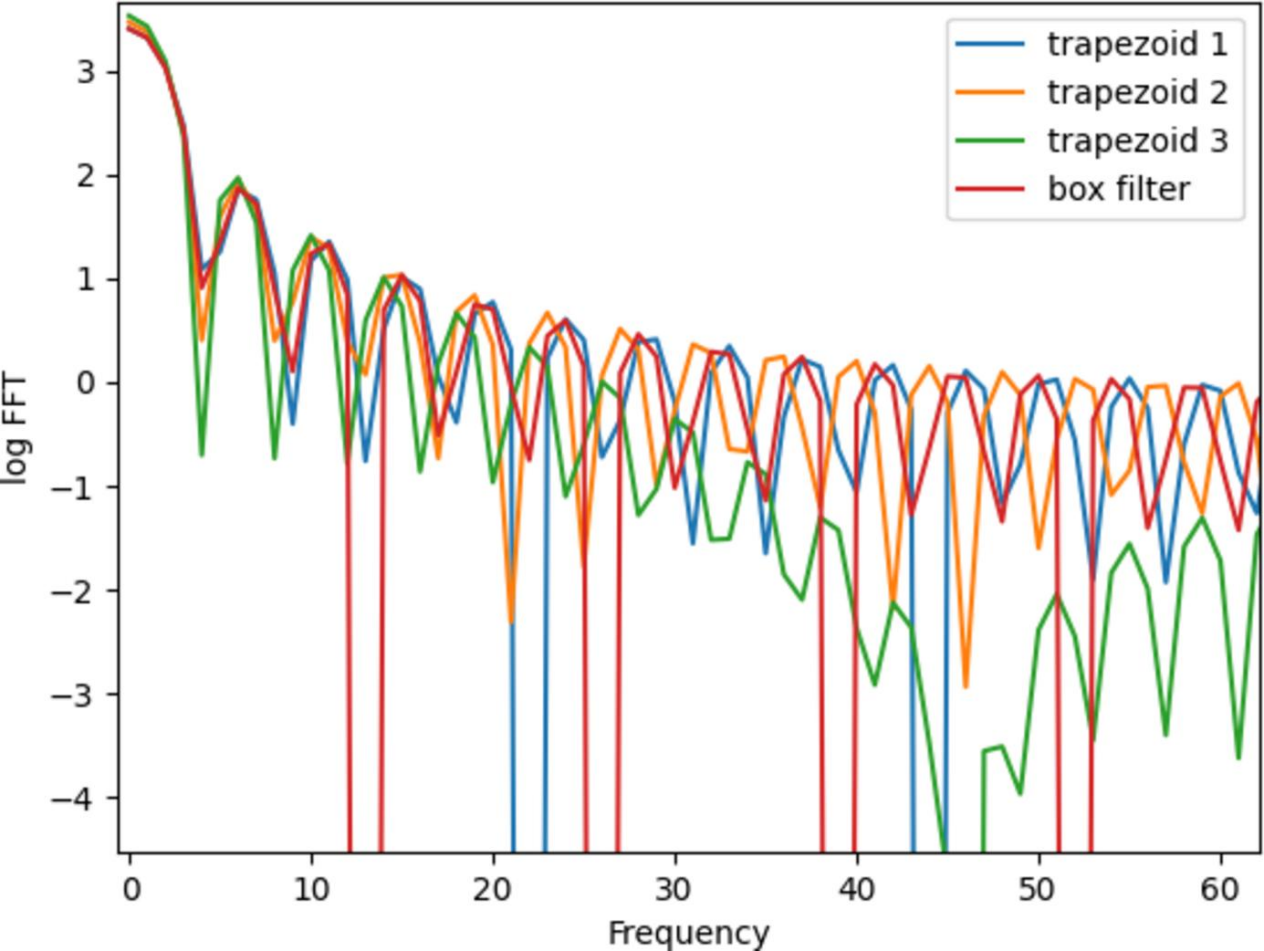
The modulus of the Fourier transform of three trapezoidal filters and a box filter.

In order to enable the deconvolution algorithm to be performed through multiple filters, the following requirement must be met: the modulus values of these filters after Fourier transform cannot have common zero-points. It is calculated that when tuning the rotation speed of the diffractometer’s arm and the collection frequency of the detector, the obtained trapezoidal filter upper and lower ratio can be set to 29:31, 7:8 and 9:11. The Fourier transform amplitude results of the three filters are shown in Fig. 2 ▸. Compared with the Fourier transform results of the red box filter, the spike positions approaching zero in the Fourier transform results of the three trapezoids do not coincide, and there is no situation in which the three Fourier transforms approach zero at the same time.

For each measurement, 

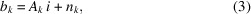

where *A*_*k*_ is the convolution kernel of the *k*th experiment, *i* is the signal result from the physical structure of the tested material, *n*_*k*_ is the noise of the *k*th measurement and *b*_*k*_ represents the result obtained by the *k*th measurement. *A*_*k*_ is an *m* × *n* matrix, *i* is a column vector of dimension *n*, and *n*_*k*_ and *b*_*k*_ are column vectors of dimension *m*. For a single measurement, if the noise term is ignored, by multiplying 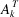 on both sides of the equation the estimated value of *i* can be obtained by solving the following equation,

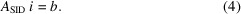

Here,

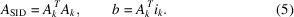

To suppress ringing artifacts and noise, some smoothing terms can be added,

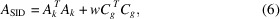

where *C*_*g*_ is the convolution matrix corresponding to the differential filter *g* = [1 −1] and *w* is the weight parameter. The value of the weight parameter should be chosen carefully and will be discussed later. For multiple images, the corresponding equation becomes *A*_MID_ *i* = *b*, where

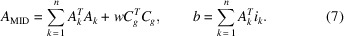

The deconvolved signal *i* can be obtained by directly solving the linear algebra equation

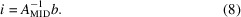




## 4. Experimental result and discussion

### 4.1. Experimental details

The experiment was carried out at beamline BL02U2 of SSRF. A Huber5021 diffractometer was used, and an Eiger 500k detector with pixel size of 75 µm. The X-ray energy was 9.85 keV, the distance between the sample and the center of the detector was 0.5 m, and the angle corresponding to the side length of the pixels was about 0.008°.

**Figure 3:**
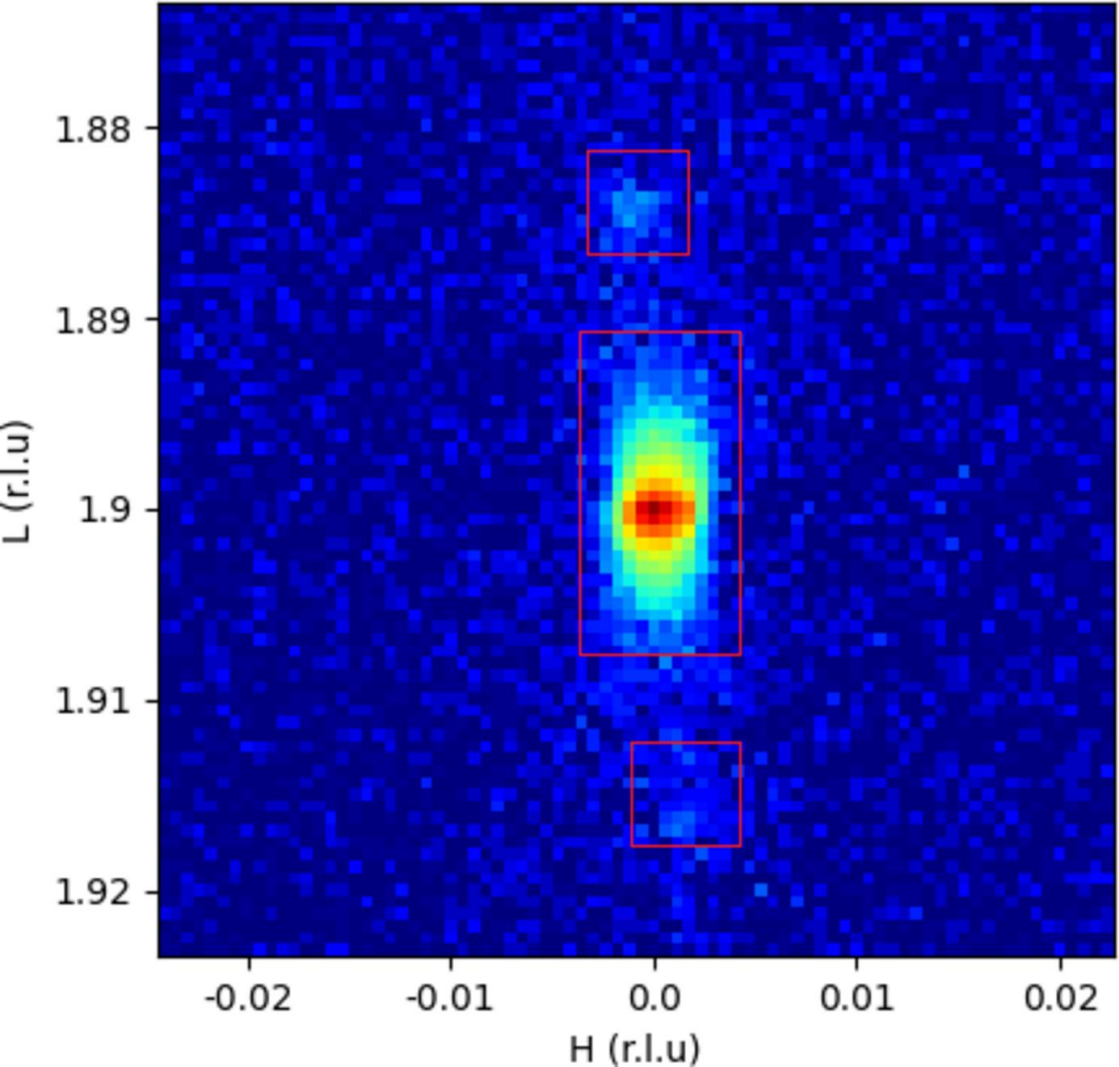
A typical result of receiving signals at the reciprocal space (0, 0, 1.9) position.

A single-crystal SrTiO_3_ (STO) substrate was chosen – the substrate surface was corroded, and this pre-treatment caused miscut on the substrate surface. This means that some sub-rods will appear in reciprocal space, and the angular distribution of these sub-rods is very narrow. Then the crystal truncation rod (CTR) signal of the substrate was measured. First, the sample orientation was adjusted, and then the position of the diffractometer’s sample stage and detector arm, so that the surface detector receives the STO substrate at the reciprocal space (0, 0, 1.9). Fig. 3 ▸ shows a typical result of received signals at this position, where the intensity is shown on a log scale. The coordinates have been transformed into reciprocal space. The sample mainly has three truncated rods signals at this position, marked by the red squares. The peak signal in the middle is slightly stronger, although it has been logarithmically processed; the upper and lower signals are still weak, especially the bottom one, whose existence is even difficult to determine.

The detector moved while collecting a series of scattered signals at high frequency. The acquisition frequency of the detector was set to 200 frames s^−1^, and the rotation speed of the arm was set to 0.028° s^−1^, 0.057° s^−1^ and 0.086° s^−1^, corresponding to the three kinds of trapezoidal filters discussed in Section 3.2. When the diffractometer’s arm moves from one position to another, the arm undergoes a process of acceleration from static to moving-at-constant-speed to deceleration to static. The subsequent procedure of data processing will discard the data from the initial and final parts and only select the data collected during the moving-at-constant-speed process in the middle. Because the collected CTR section signals are spots rather than rings, only the channels near the spots contribute to the final result, so only the channels in several columns near the spots are selected for the final angle calculation.

### 4.2. Discussion

**Figure 4:**
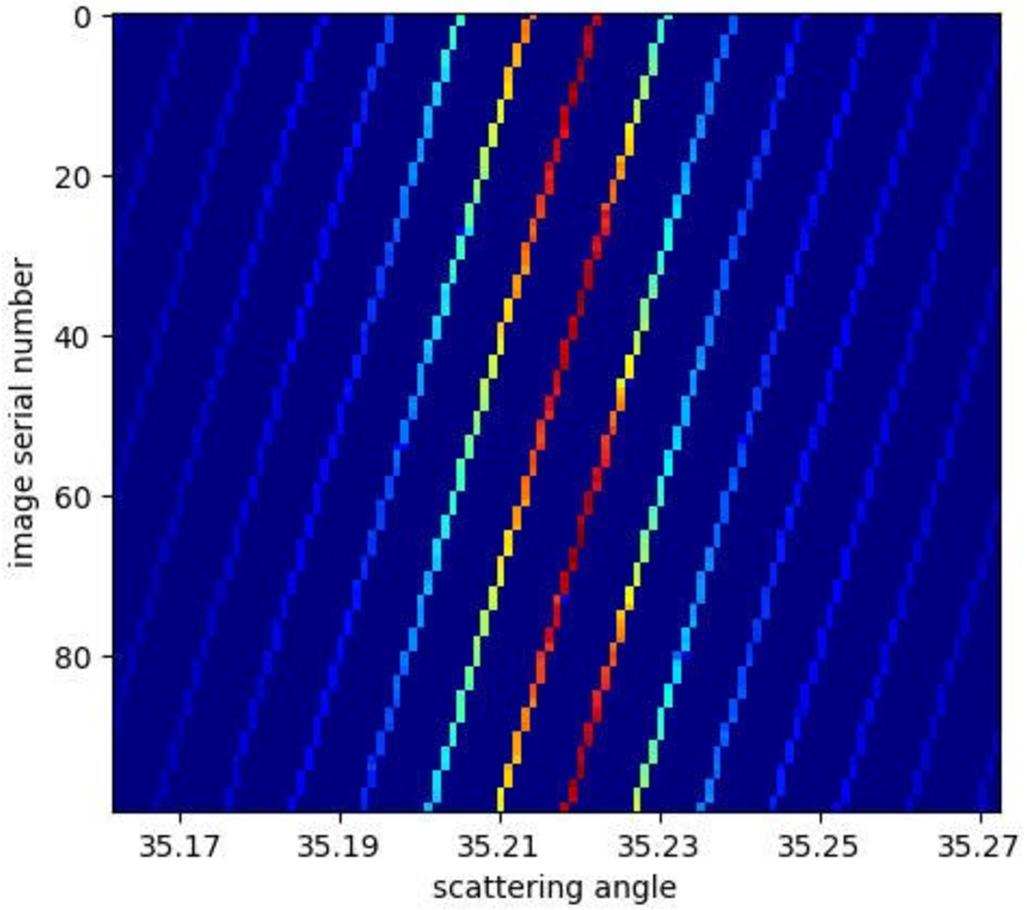
The result of angle conversion of different tested images.

As shown in Fig. 3 ▸, for the upper and lower rod sections, the distribution of the scattered X-ray signal is very narrow, resulting in a severe ‘mosaic’ pattern, with very few pixels on each peak. Such a series of images are captured for each filter. The integral of each image is converted into an angle–intensity curve, and the step of the curve is set to 0.001°, which is much smaller than the angle corresponding to the side length of a single pixel. Fig. 4 ▸ shows the result of angle conversion of different images. The horizontal coordinate is the scattering angle and the vertical coordinate is the index of the image serial number. It can be seen that, for each image (located at the same ordinate), the angle range only contains certain specific positions (where the bright lines exist), with many of the other angles not having corresponding pixels, meaning that the detector cannot obtain such a high-resolution result without the motion of the detector. Also, the strongest value of the scattered signal (deep red color) has the same interval on the vertical axis, indicating the constancy of the detector’s movement speed. It appears at the same position on the horizontal axis, indicating the accuracy of the movement speed. The results from images of all angles are aligned and superimposed. On the one hand, pixels will appear at all angles; on the other hand, thanks to the huge collection (thousands to tens of thousands of images), many pixels will be superimposed at each angle, which ultimately plays a role in data noise reduction.

**Figure 5:**
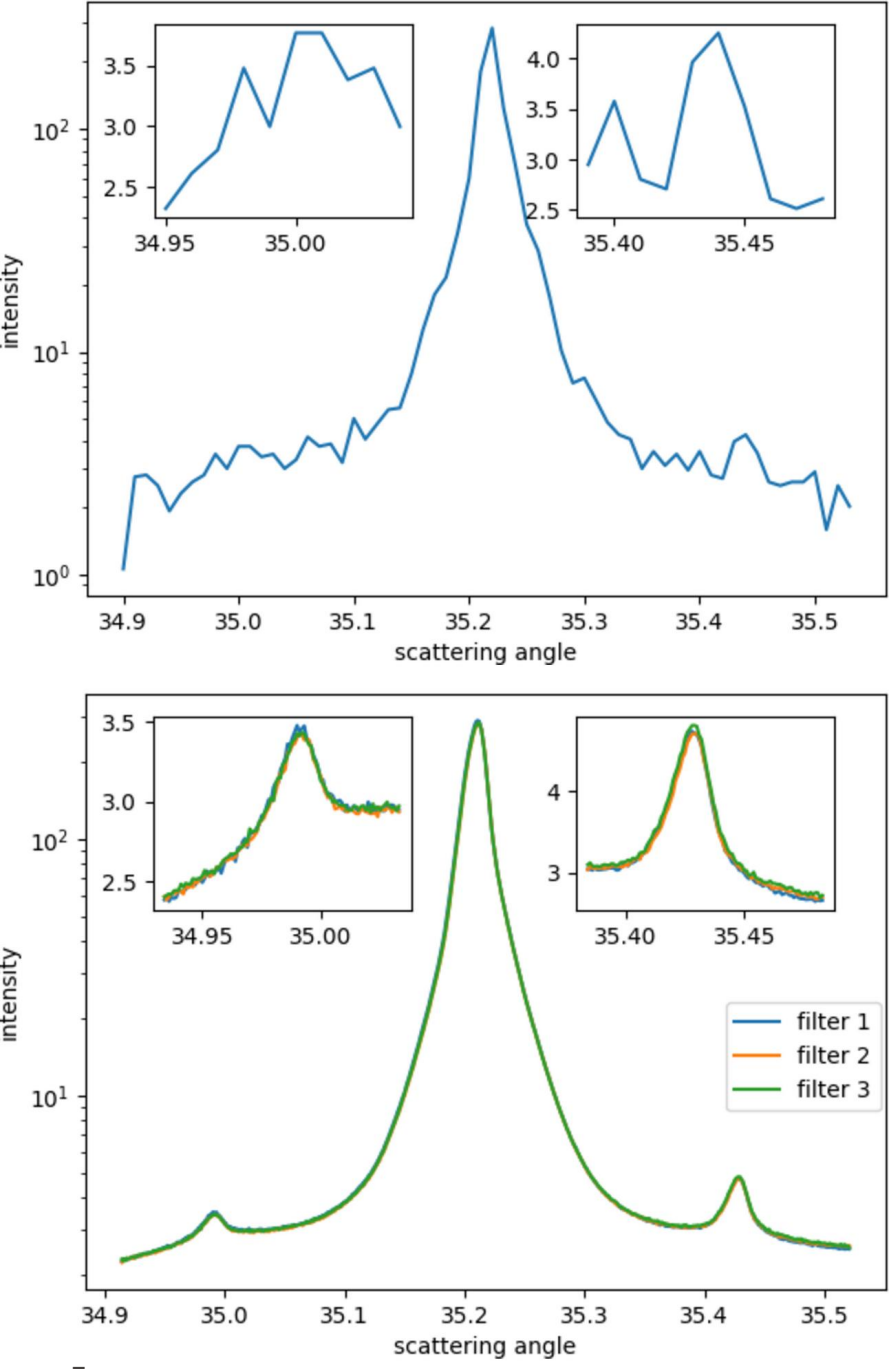
Top: results of conventional measurement methods. Bottom: results using the methodology introduced in this article.

Fig. 5 ▸ shows a comparison between the preliminary results of this method and the results of ordinary measurements; it can be seen that the signal quality is greatly improved. As mentioned above, the commonly measured angle conversion step is just slightly less than 0.01°, due to the limitation of the pixel size; if the conversion step size is reduced more, there will be many angle ranges having no pixels, thus it is impossible to achieve a very high angle resolution, so the conversion step can only be set at 0.01°. As the upper part of Fig. 5 ▸ shows, the weaker peaks in the front and back are difficult to see clearly, and it is even difficult to determine their existence, while the stronger peaks in the middle are visible. Their shape has changed greatly, caused by too few pixels at the corresponding angles. The lower part of Fig. 5 ▸ shows the final results for the three filters used in this method. It can be seen that, in addition to the strong peak in the middle, the shape of the front and back peaks is also clearly displayed. The upper part has a collection time of 5 s, while the three curves on the lower part have longer total collection times, approximately 60 s, 30 s and 20 s. This may have a certain impact on the final signal-to-noise ratio, but it can be seen that the curves of the three different acquisition times on the lower part only differ slightly. Combined with the huge difference between the upper and lower parts, it shows that the method used here may still play a certain role in noise reduction.

**Figure 6:**
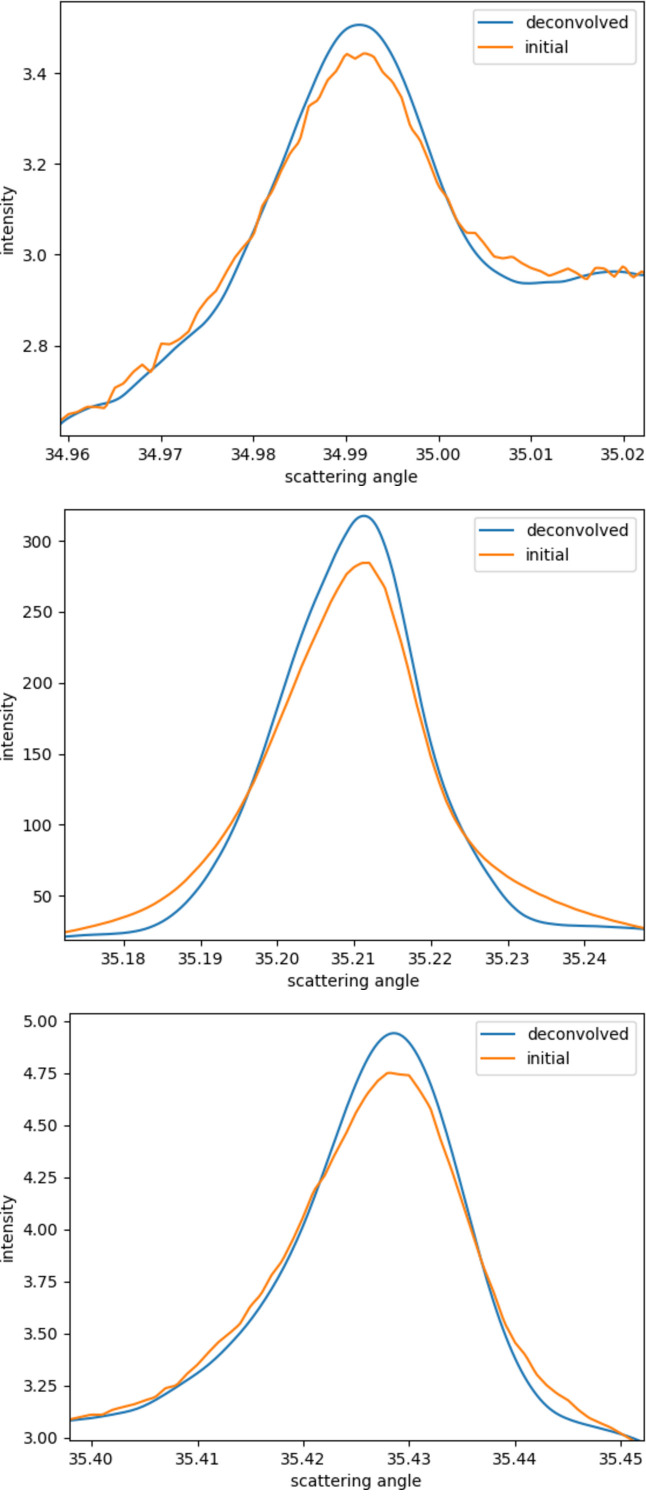
Comparison of the peak shapes of three peaks before and after deconvolution.

Fig. 6 ▸ shows the result of further deconvolution with the formula, and makes a comparison with the results without the convolution. The orange curve is obtained by averaging and interpolating the results of the three filters, and is unconvolved. The blue curve is the result of deconvolution using the previously mentioned algorithm. It can be seen that, compared with the results without deconvolution, the signal peak intensity after deconvolution is higher, and the lower trailing width is smaller, indicating that the full width at half-maximum (FWHM) will be narrower, which shows the role played by the deconvolution algorithm. In addition, the final result also shows a smoother curve, and the noise is further suppressed.

**Figure 7:**
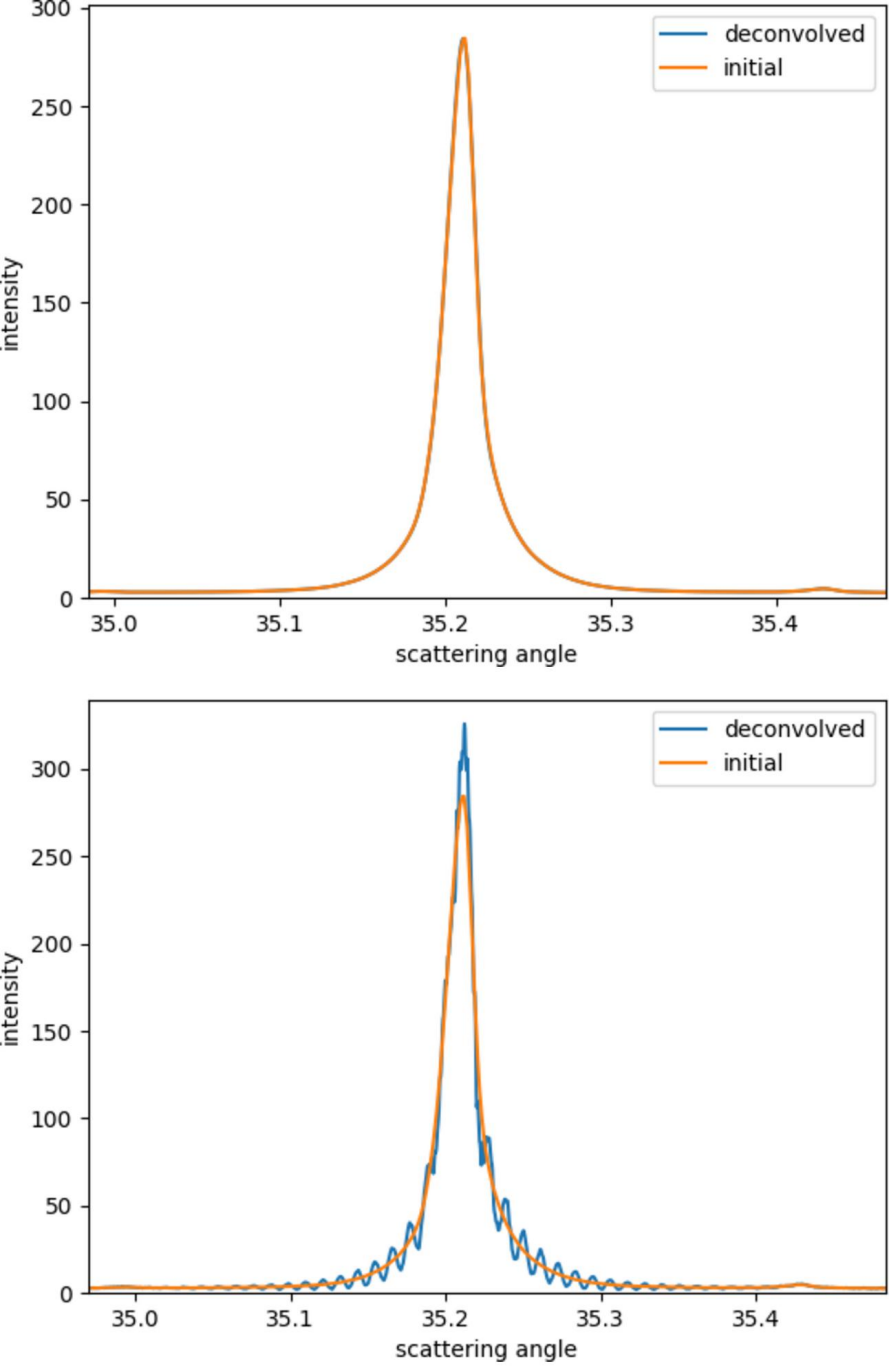
Results when the kernel size is too small (top) and too large (bottom).

All the above are calculated according to the experimental conditions. What will happen if one does not use the filter kernel with the calculated size should be discussed. As shown in Fig. 7 ▸, when the filter kernel is selected too large or too small, the result will not be ideal. When the filter kernel is too small, the result after deconvolution is very close to that before deconvolution, and it is difficult to see the deconvolution effect. When the filter kernel is selected too large, the obtained result will contain many oscillations. Only when the filter kernel is selected to match the experimental conditions are the results in good agreement. It can be proved that the selected filter kernel is reliable.

**Figure 8:**
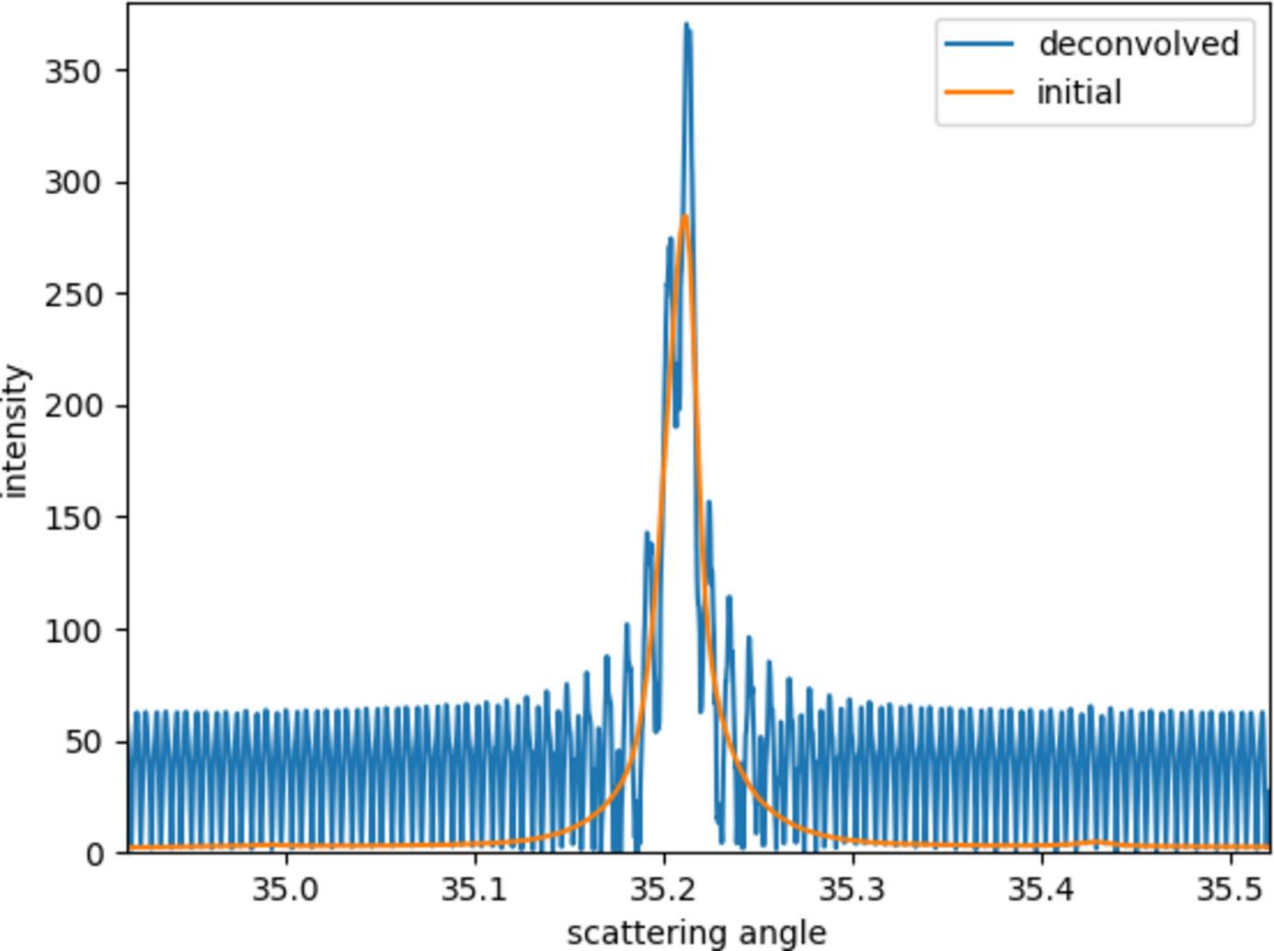
Result when the kernel size of the three filters is the same.

The purpose of using filter kernels of different lengths is to prevent the modulus of the Fourier transform from these filters having near-zero points, which will cause strong oscillations after the deconvolution operation. Fig. 8 ▸ shows the calculation result when the three filter kernels are set to the same length. It can be seen that the result will also have large oscillations when other parameters are the same. This also justified that using just one filter to obtain the deconvolved signal is impossible, and that using filters with different lengths is necessary.

**Figure 9:**
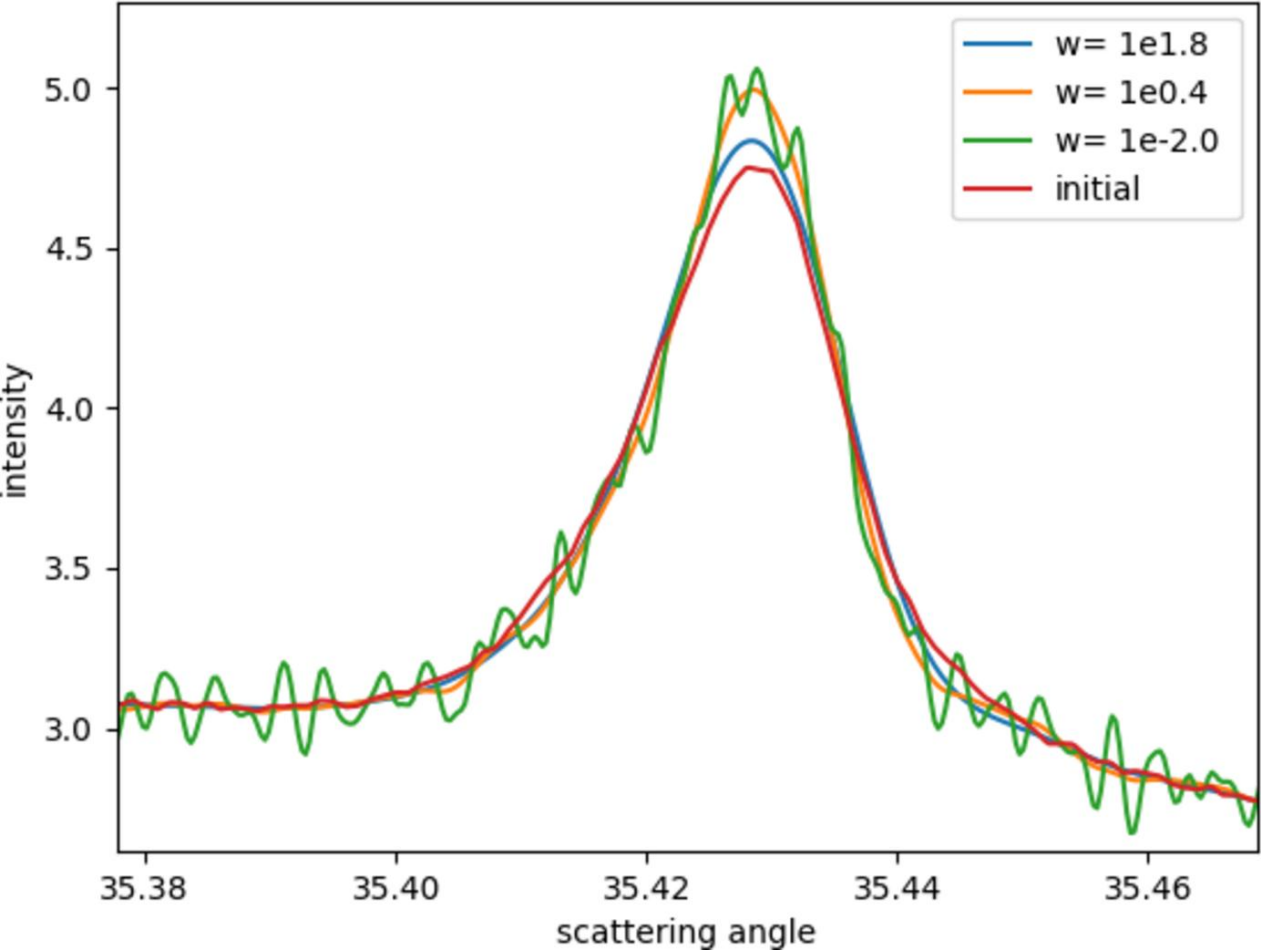
Comparison of results calculated with different smoothing coefficients.

Since smoothing terms are introduced into the formula, which will change the final results, the reliability of the results needs to be discussed. Taking the third peak as an example, it can be seen from Fig. 9 ▸ that, when the smoothing coefficient is too large (blue curve), the intensity of the curve becomes too low, whereas, when the smoothing coefficient is too small (green curve), the curve oscillation increases. When the smoothing coefficient is chosen in a certain range, the curve oscillation is neglectable, and the intensity will remain unchanged at a certain value. When the coefficient continues to decrease, the curve oscillations will increase, but the intensity does not change much, indicating that the intensity should be at a more reasonable value.

It can be seen from the discussions above that the results obtained from these filters are reasonable. In addition, using the method described in Section 3, a reasonable smoothing term coefficient can also be found to make the final result more reliable.

It is worth mentioning that in the method of collecting the signal while at the same time rotating the detector, as used here, the angular interval between two acquisitions can be far less than the motion precision limited by the diffractometer motors (one thousandth of a degree), so in theory it is hoped that the accuracy can be improved to be even better than the motion precision of the diffractometer motors.

## 5. Conclusion

We propose a method of acquiring X-ray scattered signals at a high frequency while the detector is moving. The algorithm provided in this paper can break through the limitation caused by the pixel size of the detector. The algorithm is not a common one that suppresses signals at high frequencies, ensuring the reliability of the peak shape after restoration. The feasibility of this method is verified by successfully measuring CTR signals of STO single crystal, and the resolution is improved by nearly ten times compared with that of a single pixel. In addition, this method can also suppress noise and improve the signal-to-noise ratio.
